# Infectious Diseases and Perinatal Outcomes^1^

**DOI:** 10.3201/eid1011.040623_10

**Published:** 2004-11

**Authors:** Uma M. Reddy, Alicia Fry, Robert Pass, Alessandro Ghidini

**Affiliations:** * National Institutes of Health, Bethesda, Maryland, USA;; † Centers for Disease Control and Prevention, Atlanta, Georgia, USA;; ‡ University of Alabama at Birmingham, Birmingham, Alabama, USA;; § Georgetown University, Washington, DC, USA

**Keywords:** congenital infection, listeria, cytomegalovirus, toxoplasma

## *Listeria* and Pregnancy: Trends in the United States

*Listeria monocytogenes* is a foodborne pathogen that causes life-threatening illness in approximately 2,500 persons in the United States each year, and pregnant women are particularly susceptible. Listeriosis during pregnancy can result in fetal death, premature delivery, or severe illness in the newborn. Pregnant women accounted for 16%–90% of patients in many *Listeria* outbreaks. In several outbreaks, illness occurred almost exclusively among Hispanic women who consumed Mexican-style cheese. Active surveillance in selected states from 1996 to 2000 showed a higher incidence of listeriosis among Hispanics compared with non-Hispanics, particularly in infants and women of childbearing age. Outbreak investigations identify *Listeria*-contaminated food items, facilitate their removal from the market, and identify problems in food processing methods. This information has been instrumental in developing Food and Drug Administration and United States Department of Agriculture policies to reduce *Listeria* contamination of foods and in motivating industry interventions. Listerioisis can be prevented by encouraging pregnant patients to avoid soft cheeses, unless labeled as made from pasteurized milk; hot dogs, luncheon meats, or deli meats, unless they are reheated until steaming hot; refrigerated pâtés or meat spreads; and refrigerated, smoked seafood (http://www.cdc.gov/ncidod/dbmd/diseaseinfo/listeriosis_g.htm). Special efforts to develop educational tools for pregnant Hispanic women are currently underway.

## Congenital CMV and Toxoplasmosis: Outcome for the Infected Newborn

Data on the outcome of congenital infections caused by cytomegalovirus (CMV) and by *Toxoplasma gondii* are reviewed. CMV is the most common cause of congenital infection in the United States, occurring in ≈1 in 40,000 live-born infants per year, and is ≈100 times more common than congenital toxoplasmosis. Each of these infections is important in reproductive health because of its ability to infect the fetus and cause damage to the central nervous system, including visual and auditory organs. Maternal gestational infection with either agent is likely to be asymptomatic and to result in transmission to the fetus in ≈20% to 50% of cases. Although the two infections can have substantial overlap in both newborn findings and sequelae, congenital CMV infection is most noted as a cause of hearing loss and mental retardation, while congenital toxoplasmosis is known for its association with chorioretinitis, visual impairment, hydrocephalus, and mental retardation. CMV appears to be the leading cause of sensorineural hearing loss in young children. Large studies on the outcome of congenital toxoplasmosis performed in the past showed that few untreated patients (10%–15%) escaped central nervous system sequelae.

Antimicrobial drug treatment of infants with congenital toxoplasmosis is recommended by specialists in the field and accepted as standard of care. Although no randomized, double-blind, placebo-controlled clinical trials demonstrate efficacy of such treatment, comparing outcomes of treated patients with those of historic, untreated cohorts provides evidence that supports the effectiveness of treatment. Treatment of congenital CMV infection is not as widely accepted. Treating newborns with asymptomatic congenital CMV infection is not recommended because of their low (10%–15%) risk of sequelae. Antiviral treatment of maternal CMV infections with the aim of preventing or ameliorating fetal infection has not been evaluated.

Although treating maternal gestational *Toxoplasma* infections has been advocated by experts for more than a decade, results from several more recent European studies did not show that maternal treatment reduced transplacental transmission. Whether treating the infected fetus (after prenatal diagnosis) will reduce the severity of the congenital infection is controversial, although many authorities recommend such treatment.

Public health efforts aimed at preventing *Toxoplasma* infection associated with maternal environment or food exposures can be effective in reducing infection rates. Because the sources of maternal CMV infection are ubiquitous and difficult to avoid, preventing congenital CMV infection will likely require development of an effective vaccine.

